# Susceptibility to Size Visual Illusions in a Non-Primate Mammal (*Equus caballus*)

**DOI:** 10.3390/ani10091673

**Published:** 2020-09-17

**Authors:** Anansi Cappellato, Maria Elena Miletto Petrazzini, Angelo Bisazza, Marco Dadda, Christian Agrillo

**Affiliations:** 1 Department of General Psychology, University of Padova, 35131 Padova, Italy; anansi.cappellato@studenti.unipd.it (A.C.); angelo.bisazza@unipd.it (A.B.); marco.dadda@unipd.it (M.D.); 2 Department of Biomedical Sciences, University of Padova, Via Bassi 58, 35131 Padova, Italy; mariaelena.milettopetrazzini@gmail.com; 3 Padua Neuroscience Center, University of Padova, Via Orus 2, 35131 Padova, Italy

**Keywords:** visual illusions, cognitive ethology, comparative perception

## Abstract

**Simple Summary:**

Visual illusions are commonly used by researchers as non-invasive tools to investigate the perceptual mechanisms underlying vision among animals. The assumption is that, if a species perceives the illusion like humans do, they probably share the same perceptual mechanisms. Here, we investigated whether horses are susceptible to the Muller-Lyer illusion, a size illusion in which two same-sized lines appear to be different in length because of the spatial arrangements of arrowheads presented at the two ends of the lines. Horses showed a human-like perception of this illusion, meaning that they may display similar perceptual mechanisms underlying the size estimation of objects.

**Abstract:**

The perception of different size illusions is believed to be determined by size-scaling mechanisms that lead individuals to extrapolate inappropriate 3D information from 2D stimuli. The Muller-Lyer illusion represents one of the most investigated size illusions. Studies on non-human primates showed a human-like perception of this illusory pattern. To date, it is not clear whether non-primate mammals experience a similar illusory effect. Here, we investigated whether horses perceive the Muller-Lyer illusion by using their spontaneous preference for the larger portion of carrot. In control trials, we presented horses with two carrot sticks of different sizes, and in test trials, carrot sticks of identical size were shown to the subjects together with arrowheads made of plastic material and arranged in a way meant to elicit the Müller-Lyer illusion in human observers. In control trials, horses significantly discriminated between the smaller and larger carrot stick. When presented with the illusion, they showed a significant preference for the carrot that humans perceive as longer. Further control trials excluded the possibility that their choices were based on the total size of the carrot stick and the arrowheads together. The susceptibility of horses to this illusion indicates that the perceptual mechanisms underlying size estimation in perissodactyla might be similar to those of primates, notwithstanding the considerable evolutionary divergence in the visual systems of these two mammalian groups.

## 1. Introduction

Understanding how animals see the world is one of the main issues of cognitive ethology. To achieve this goal, the neurobiological investigation of photoreceptors and the study of neural circuits of vision are fundamental to delineate which information is captured by the retina and how this information is processed in the brain. However, this type of investigation gives limited insight into how each animal sees the surrounding world. With respect to this issue, visual illusions represent a unique opportunity for understanding the perceptual laws that underlie vision in humans and in other species. The study of visual illusions helps us to shed light on sensory information processing modes, in terms of bottom-up (how the final perception is built based on sensory inputs) and top-down (which knowledge stored in the brain shapes the perception of the visual scene) mechanisms [1,2].

Size illusions represent one of the largest categories of visual illusions. They occur when individuals misperceive the size of an object as a function of the context (e.g., the surrounding background). Three popular visual illusions belong to this category: the Ponzo illusion (two same-sized lines appears to be different based on their background), horizontal–vertical illusion (a vertical line appears longer than a horizontal one even if they are identical in size), and Muller-Lyer illusion (two same-sized lines, one of which ends in inward pointing arrows and the other which ends with outward pointing arrows, appear to be different in length). All these illusions seem to be due to the tendency to extrapolate inappropriate 3D information from 2D patterns [3,4]. For instance, in the case of the Muller-Lyer illusion, the line with the arrowheads pointing inward would appear farther than the line with the arrowheads pointing outward. Because of the size-constancy mechanisms commonly involved to compensate for the decreasing retinal size of an item with increasing distance, the former line would appear longer.

The susceptibility to Muller-Lyer illusion has been reported in both Old and New World monkeys [5,6], suggesting that the ‘inappropriate size-scaling’ may characterize the visual perception of all simian primates. To date, it is unknown how other mammals perceive this illusion. The only non-primate mammal studied so far is the dog, which does not perceive size illusions in the same way as primates [7]. Like most carnivorans, canids are primarily nocturnal or crepuscular species and their surrounding space is largely sensed through hearing and olfaction. Size illusions might therefore be the consequence of the acquisition of an accurate depth perception and the evolution of a sophisticated system of three-dimensional vision that has occurred during primate evolution following the adaptation to arboreal habitat and diurnal life. The sample of species studied so far is obviously insufficient to reach any conclusion. More mammalian species need to be examined, in particular extending the investigation of size visual perception to other well studied mammalian orders such as rodents, odd-toed and even-toed ungulates.

Human–horse interaction, dating back to approximately 6000 BC [8], represents a long-term relationship, the significance of which is amplified by the key role that horses have played in different fields of human economy. Given the relevance of horses to productivity in human society, it is not unexpected that the visual capacities of this species are well studied [9,10]. The attention of researchers has been particularly focused on colour perception [11], depth perception [12], visual acuity [13], monocular/binocular visual fields [14,15], scotopic vision [16], and interocular transfer [17]. In addition, Timney and Keil [18] found that horses are sensitive to pictorial depth cues. In this study two subjects were trained to select the longer line between two lines placed one above the other. In the test phase, two same-sized lines were superimposed on a photograph of railway tracks. Horses selected the line close to the converging track rails, showing to use pictorial depth cues to infer the length of the two lines. Investigating a species that is less reliant on vision, whose visual system largely differs from that of primates, would be particularly useful to address the issue as to whether the perception of size illusions is related to the evolution of stereoscopic vision and the development of sophisticated systems for reconstructing three-dimensional information.

Here, we investigated the extent to which horses demonstrate sensitivity to the Muller-Lyer illusion. There are multiple evidences that horses exhibit quantificational abilities. Horses can discriminate the larger group of food items [19] or the larger set of dots in a bi-dimensional figure [20]. Also, they are capable of discriminating between continuous quantities. Hanggi [21] showed that horses, initially trained to select the larger stimulus between two two-dimensional figures, are able to generalize the learned rule to both novel 2D stimuli and 3D configurations, showing a true concept of size. The precision of such size discrimination can be very high, with horses being able to discriminate up to 14% difference in objects’ size [22].

We adapted a procedure used in research on other mammals [23,24,25,26] that consists of the observation of the spontaneous preference of the animals for reaching the largest amount of food. In control trials, we presented subjects with two portions that differed in size. Horses were expected to maximize the amount of food to be ingested. In test trials, two identical portions of food were arranged to generate the experience of the Müller-Lyer illusion in human observers. If horses were susceptible to the illusion, we expected them to select the food portion with the inward-pointing arrows.

## 2. Methods

We preliminarily assessed the sample size using Fisher’s exact test, choosing α = 0.05 and a desired power of 0.80. Assuming a minimal detectable difference in means in the proportion of choices of 0.097 as and a standard deviation of 0.09 (data taken from our pilot experiments), we found that a total of 9 subjects was appropriate to test our hypothesis. Accordingly, we tested 10 domestic horses (*Equus caballus*, average age 11.4; 1:1 male:female; see Table 1 for age, breed, and sex of each individual subject). Subjects were housed at a private riding stable (Scuderia San Martino) located in Legnaro (Padova, Italy), where we conducted the experiments. The daily diet included hay ad libitum and specific animal feed. They were also fed with fresh vegetables (including carrots) and fruits weekly. All subjects were experimentally naive before the beginning of our study. 

**Figure 1:**
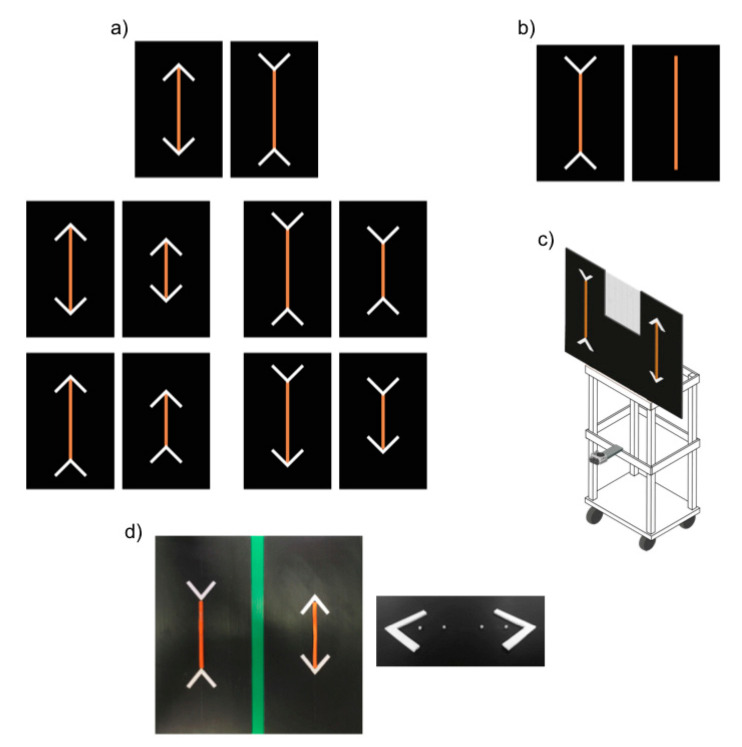
Stimuli used in the study. (**a**) The Müller-Lyer illusion and the four different configuration types used in the control trials; (**b**) stimuli used in the control test for overall size; (**c**) simulation of the placement of stimuli on the tray; (**d**) picture of the stimuli (**left**); arrowheads and plastic screws used to hold the carrot sticks (**right**).

The experimental apparatus consisted of a wooden tray (45 × 37 × 105 cm) supporting a black plastic panel (90 × 61 cm) on which stimuli were presented (see Figure 1c). A window (22.5 × 39 cm) delineated by a black net (1 × 1 mm grid pattern) was present in the upper part of the panel, enabling the experimenter to observe the position of the horse prior to presentation of the stimuli while rendering the experimenter invisible to the subject horse. A video camera (GoPro Hero4) placed below the panel was used to record the experiment.

The stimuli consisted of carrot sticks arranged on the above described black plastic panel. A series of four small plastic screws permits to fix the carrots in the panel (Figure 1d). In addition, four white plastic sticks (7 × 1 × 1 cm) were arranged to form two arrowheads on the two sides of each carrot stick. We used arrowheads that differed in colour and texture from the carrot sticks to ensure horses could easily distinguish between food and non-food items, thus focusing on the longer food portion and not on the overall longer configuration made by the food and the arrowheads. The use of different colours between lines and arrowheads is commonly adopted in comparative studies on Müller-Lyer illusion to make the subject focus on the main line [27,28].

In control trials, the assumption that subjects would aim to maximize food intake was tested. For this purpose, subjects were presented with a larger (20 × 1 × 1 cm) and a smaller (13.5 × 1 × 1 cm) carrot stick (removed from the top and the bottom of the carrot) and allowed to make a choice. The size ratio of the two portions was 0.675, in terms of both length and volume. The control trials served an additional purpose of familiarising subjects with the presence of the carrot sticks and the arrowheads in preparation for the test trials. In particular, to ensure the horses got accustomed to changes in orientation of the arrowheads, in control trials subjects were presented with 4 disparate configuration types (Figure 1a):

Control A: Larger vs. smaller food portion, with all arrowheads pointing inward, symmetrically oriented to diverge at 135° angles from the food portion.

Control B: Larger vs. smaller food portion, with all arrowheads pointing outward, symmetrically oriented to diverge at 45° angles from the food portion.

Control C: Larger vs. smaller food portion; for each portion, one arrow pointed inward (the one presented below, at 135°) and the other outward (above, at 45°).

Control D: Larger vs. smaller food portion; for each portion one arrow pointed inward (the one presented above, at 135°) and the other outward (below, at 45°).

In test trials, subjects were presented with two illusory patterns made up of two same-sized food portions (20 × 1 × 1 cm). In one array the two arrowheads pointed inward (135°), while in the other they pointed outward (45°). In the presence of such patterns, human observers typically estimate the former array to be the larger.

The experiment was conducted in the box (12 m^2^) wherein subjects were housed. The box was provided with a 120 × 250 cm stable door that can be turned into a 120 × 120 cm window. At the beginning of each day, 4 preliminary trials were conducted to assess the extent to which the horses were motivated to reach the food and whether they exhibited any side biases. In each trial, only a single carrot stick (average size: 6 cm) was presented on one side of the panel (to either the left or the right). If the subject did not reach for the food item when it was presented on both sides (e.g., a trial on the right, another trial on the left; 4/4 choices for the side presenting the carrot), no experimental test session was scheduled for that day. In these circumstances, preliminary trials were presented again on the following day to determine whether the subject could commence participating in the daily session.

**Figure 2:**
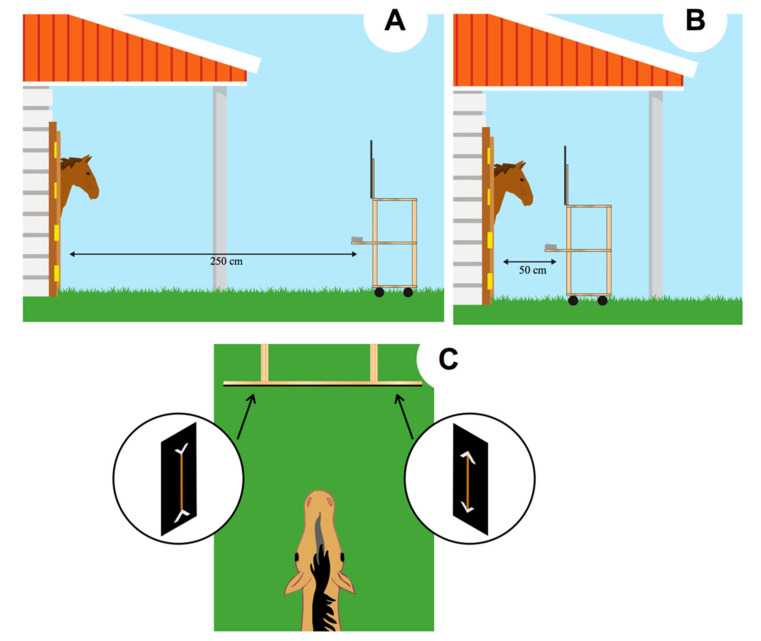
Experimental procedure. The tray was initially placed at a distance of 2.5 m (**A**). Then, the tray was pushed from behind toward the subject until it reached a distance of 50 cm (**B**). The horses were free to select one of the two display boards, by eating one of the two carrots (**C**).

The experiment consisted of the daily presentation of every condition. Horses not involved in the training session could not see the performance of the tested horse as the adjacent boxes were kept close during the trial. The tray was initially placed at a distance of 2.5 m in front of the box (Figure 2A). As soon as the stimuli were attached to the display out of the horse’s view, the tray was gently pushed from behind toward the subject until it reached a distance of 50 cm from the subject (Figure 2B). In this way, the horse had the opportunity to clearly see the stimuli, both when the stimuli were far (e.g., by both binocular and monocular view) and when they were near the subject. Horses have a blind spot, triangular in shape, up to 120 cm in the central part of the binocular visual field. However, the size of this blind spot seems to be determined by the level at which horses’ head is carried: the smallest blind area is supposed to be obtained when the horses’ head point down [29], as the case of our experimental set up (the tray was lower than the horses’ head). In addition, the inter-item distance was 60 cm (similar to the inter-item distance used by Tomonaga et al. [22] in their size discrimination task), a fact that should have minimized the possibility that, at the time of the choice, horses cannot see the stimuli.

The experimenter’s body was not visible to the horse as it was entirely covered by the black plastic panel. Always the same experimenter presented the stimuli. The horse spontaneously selected one of the two display boards, by eating one of the two carrots presented (Figure 2C). The responses were unambiguous. Each trial lasted, on average, 5–7 s. As soon as the horse selected one stimulus, the tray was pulled back to avoid the possibility to reach also the other carrot. Each horse’s selection was observed by the experimenter via a smartphone, paired with the camera placed below the plastic panel presenting the stimuli. Horses participated in twelve daily sessions. Each session started at 8.00 am and consisted of 6 trials, over 12 days, for a total of 72 trials. Sessions consisted of 4 control trials (one of each type) and 2 test trials. The order of presentation of the different types of trials varied following a pseudo-random sequence. The position (left/right) of both the large and the small portion was counterbalanced across the trials to inhibit side biases.

Based on our experimental results, we set up a control test for the overall size of the stimuli recently used in the study on dogs [7]. Indeed, horses in the previous test phase overwhelmingly selected the food portion with the inward-pointing arrows. This result could have been due to two reasons: (a) horses were sensitive to the Müller-Lyer illusion or, (b) in the presence of the illusory pattern, they might have identified a discriminative cue, such as a physical difference between the two arrays (i.e., the one with the inward-pointing arrowheads occupied a larger space, overall, than its inverse). As such, we presented a larger carrot (30 × 1 × 1 cm) relative to a smaller one (20 × 1 × 1 cm). The overall configuration of the smaller carrot, however, was identical in overall size (30 cm), relative to the other stimulus, as the inward-pointing arrowheads were included (Figure 1b). The control test, for the overall size, involved 18 trials (3 sessions, each consisting of 6 trials).

## 3. Results

We conducted a linear mixed model (LMM) with condition (Control A/B/C/D/Illusory trials/Control for overall size) as within-subjects factors on the proportion of choice for the physically (control trials) or subjectively larger (test trials) carrot stick that revealed a significant effect of the Condition (F(5, 54) = 3.367, *p* = 0.010) on the horses’ performance. A Tukey post-hoc test showed that such difference is only due to a statistical difference between Control A and the Illusory trials (*p* = 0.008; all other *p* > 0.05). The test of the random-effect term of the LMM revealed that the subjects did not statistically differ (*p* = 1.000), thus showing no inter-individual differences. No difference in the proportion of choices was observed as a function of the experimental sessions (generalized linear mixed-effects χ2(11) = 11.083, *p* = 0.436).

Horses proved able to discriminate among all types of control trials: Control A, one-sample *t*-test: *t*(9) = 9.462, *p* < 0.001, Cohen’s d = 2.992; Control B: *t*(9) = 7.619, *p* < 0.001, d = 2.409; Control C: *t*(9) = 8.159, *p* < 0.001, d = 1.865; Control D: *t*(9) = 5.776, *p* < 0.001, d = 1.827. In test trials, horses selected the food portion presented with inward-pointing arrows more frequently than they might by chance, *t*(9) = 6.626, *p* < 0.001, d = 2.095 (Figure 2). This conclusion is also supported by Bayesian analysis: we calculated an approximate Bayes factor for one sample t-tests enabling the relative strength of the evidence for the two competing hypotheses to be estimated [30]. The approximate Bayes factor indicated that the probability that horses select the stimulus that appears to be larger by human observers (test trials) is 294 times more likely than the alternative hypothesis.

**Figure 3:**
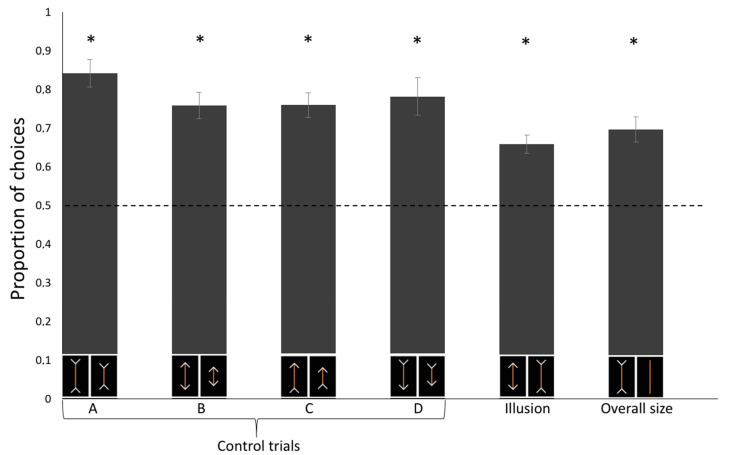
Proportion of correct choices plotted against the four control conditions (**A**–**D**) and the control for the overall size. In the test phase, the *y*-axis refers to the proportion of selections of carrots with inward-pointing arrowheads. Asterisks denote a significant departure from chance (*p* < 0.05). Error bars represent the standard errors.

In the control test for overall size, we found a significant preference for the larger food portion without arrowheads, *t*(9) = 6.024, *p* < 0.001, d = 1.905 (Figure 3).

## 4. Discussion

This study provides novel evidence that horses perceive visual illusions similarly to humans. In test trials, involving presentation of the illusory pattern, horses were found to select the food portion with the inward-pointing arrowheads, indicating a human-like perception of the Müller-Lyer illusion. The fact that horses selected the stimulus perceived as larger by human observers also indicated that horses, during the experiment, did not rely on olfactory cues to solve this task, because olfactory information coming from two same-sized sticks was presumably identical. One may argue that the performance shown in test trials might not reflect a subjective misperception of size but that the horses were attending to the totality of the configuration (food portion plus the arrowheads) instead of focusing on the two carrot sticks only. The result of control tests ruled out this alternative explanation, leading to the conclusion that horses are indeed sensitive to size visual illusions. The only significant difference between control and test (illusory) trials was found in the comparison between Control A and illusory trials. Unfortunately, we can only speculate on this result. The illusory pattern is expected to generate subtle differences in the perception of length of the two stimuli, hence it is not surprising that the performance appears to be slightly below to that observed in a control condition with a 0.675 ratio between the stimuli. In addition, it is worth noting that Control A is the condition in which the two stimuli occupy, overall, the larger space (carrot + arrowheads) compared to all other control conditions. It is possible that the relative difference between the two carrot sticks was more evident in the present of these large stimuli compared to the illusory patterns.

Our results of a similarity in response to the visual size illusion of horses and primates are particularly interesting in view of the large differences in both their ecological adaptations and their visual system. While primates live in complex three-dimensional environments, the typical habitat in which the ancestor of modern domesticated horses evolved is the Eurasian steppe, an almost bi-dimensional environment [31]. The horse has a fairly well-developed visual system, but it also strongly relies on other sensory modalities to acquire information from the external world [32]. The visual systems of humans and horses differ significantly in many respects, including a greater development of binocular vision in humans and inferior visual capabilities in horses compared to humans in terms of acuity, accommodation, and daily colour vision (reviewed in [9,10]). Our finding that horses are sensitive to the Müller-Lyer illusion is relevant to this issue as it indicates that, despite the wide range of neuroanatomical differences between the visual systems of humans and horses, there are interesting parallels in the perceptual mechanisms underlying representation of the visual scene. It is widely agreed that, in humans, the illusion operates on the basis of a misapplication of size constancy scaling [3]. In everyday life, size constancy allows us to perceive objects in a stable manner, even when the viewer’s distance from the objects changes [33]. This principle is inappropriately applied to two-dimensional objects, producing results that reflect the perception of the stimulus with the inward-pointing arrowheads as farther than the one with the outward-pointing arrowheads. Given that the two stimuli are physically identical as retinal images, the brain would thereby be subjectively enlarging the length of the stimulus that appears farther. This mechanism could potentially account for the response of the horse to Müller-Lyer illusion and explain why dogs and horses differ in this respect. Indeed, there is good evidence that horses, like humans and non-human primates, are sensitive to depth information contained in static pictures (Guinea baboon: [34]; horse [18]) while dogs use mainly other clues to estimate distance and show little sensitivity if any to pictorial depth cues [35,36].

At the present state of the research it is not possible to understand if the response to size illusory patterns was inherited in primates and ungulates from a common ancestor or it represents a by-product of habitat-driven evolution of analogous mechanisms of depth perception. Few other species have been so far investigated for their response to Müller-Lyer illusion. The three species of birds studied [27,37,38] are all diurnal, aerial, or arboreal, and all see the illusion in a primate-like way. Of the three species of fish studied, two are diurnal freshwater teleost [28,39] that have a well-developed vision and live in a three-dimensional habitat whose complexity is comparable to the arboreal habitat of primates and birds. Both species perceive this size illusion as primates and birds do. The third species, the bamboo shark [40], does not appear to perceive size illusions. This is a mainly nocturnal bottom-dwelling species that has a poor vision and, like dogs, relies mostly on other sensory modalities to sense the surrounding world.

Clearly, data from many more species need to be collected to unravel these questions, especially selecting organisms from a variety of habitats and with different visual adaptations.

## 5. Conclusions

We provided the first evidence that horses perceive the Muller-Lyer illusion, a fact that suggests similar perceptual mechanisms between primates and horses in the estimation of objects’ sizes.

## Figures and Tables

**Table 1:** Information about subjects.

Subject	Sex	Breed	Age
Kaprice	Male	Sella italiano	5
Quando	Male	Sella italiano	15
Caramella	Female	Pony	21
Castiel	Male	Sella italiano	6
Zonne	Female	Holland	14
Olvier	Male	Pony	14
Fog	Male	Anglo-arabian	16
Alissa	Female	Quarter	10
Shea	Female	Quarter	7
Spring	Female	Quarter	6
